# Importance of high-quality evidence regarding the use of Bacopa monnieri in dementia

**DOI:** 10.3389/fnagi.2023.1134775

**Published:** 2023-03-01

**Authors:** Ayush Agarwal, Biswamohan Mishra, Anu Gupta, M. Vasantha Padma Srivastava, Aneesh Basheer, Jyoti Sharma, Venugopalan Y. Vishnu

**Affiliations:** ^1^Department of Neurology, All India Institute of Medical Sciences, New Delhi, India; ^2^DM Wayanad Institute of Medical Sciences (DM WIMS), Wayanad, India

**Keywords:** Bacopa monnieri, Brahmi, dementia, Alzheimer’s disease, evidence based medicine, mild cognitive impairment, high-quality evidence

## Abstract

**Background:**

Bacopa monnieri (BM), a commonly used herb, has shown neuroprotective effects in animal and *in vitro* studies; but human studies on patients with Alzheimer’s Disease (AD) have been inconclusive. Further high-quality trials are required to conclusively state the utility of BM in AD and other neurodegenerative dementias.

**Methods:**

In the present study, we did a narrative review of the current challenges in designing clinical trials of BM in dementia and their evidence-based recommendations.

**Results:**

Many facets of the BM trials need improvement, especially effect size and sample size estimation. Current assessment and outcomes measures need a more holistic approach and newer scales for diagnosing and monitoring prodromal AD. The stringent guidelines in CONSORT and STROBE are often considered difficult to implement for clinical trials in ayurvedic medications like BM. However, adherence to these guidelines will undoubtedly improve the quality of evidence and go a long way in assessing whether BM is efficacious in treating AD/prodromal AD patients and other neurodegenerative dementias.

**Conclusion:**

Future studies on BM should implement more randomized controlled trials (RCTs) with an appropriate sample size of accurately diagnosed AD/prodromal AD patients, administering a recommended dosage of BM and for a pre-specified time calculated to achieve adequate power for the study. Researchers should also develop and validate more sensitive cognitive scales, especially for prodromal AD. BM should be evaluated in accordance with the same rigorous standards as conventional drugs to generate the best quality evidence.

## 1. Introduction

Ayurveda is an ancient system of medicine that originated in India over 3,000 years ago. It has recently gained popularity, especially as more people seek natural and holistic approaches to health and wellness. Despite the growing popularity of Ayurveda, it still faces many challenges in establishing its place as a mainstream form of medicine. One of the key obstacles is the lack of high-quality evidence to support its effectiveness. This is because many clinical trials of Ayurvedic treatments have not been conducted to rigorous standards, making it difficult to determine their safety and efficacy.

Some of the issues facing clinical trials of Ayurveda include:

• Lack of standardization: There is a lack of standardization in the manufacturing process and formulation of Ayurvedic medicines, leading to difficulties in reproducibility and consistency in clinical trials.
• Determining efficacy: Ayurvedic treatments are often complex and involve multiple ingredients, making it challenging to determine the specific component responsible for the therapeutic effect.
• Placebo effects: Patients in Ayurvedic trials often expect a positive outcome, leading to a high placebo response rate that can impact the interpretation of trial results.
• Regulatory challenges: Ayurvedic treatments are often not regulated as drugs, and there is a lack of clear guidelines for conducting clinical trials, making it difficult to obtain regulatory approval.
• Cultural and language barriers: Ayurvedic treatments are often deeply rooted in Indian culture and language, making it challenging for Western researchers to understand and conduct trials.

These challenges make it difficult to determine the safety and efficacy of Ayurvedic treatments and to establish their place in modern medicine. It is important to note that these challenges are not limited to one particular herb or treatment, but are present across the field of Ayurveda. In order to establish the place of Ayurveda in modern medicine and ensure its safe and effective use, it is necessary to bring more standardization, clarity, and scientific rigor to its practices. This can be achieved through continuous research and the development of rigorous and well-designed clinical trials, which will help to understand the mechanisms of action better and determine the safety and efficacy of Ayurvedic treatments. Additionally, regulatory bodies can play a crucial role in establishing standards and guidelines for the practice of Ayurveda, and ensuring that patients receive safe and effective treatments (Rastogi, 2010, 2011, 2018).

It is also important to educate the public on the correct identification of herbs and their possible use and adverse events. Misidentification can lead to the consumption of potentially harmful substances, which can have serious health consequences. In addition, it is essential that healthcare practitioners, including Ayurvedic practitioners, provide accurate and evidence-based information to their patients and the public to ensure that they are making informed decisions about their health. The COVID-19 pandemic has highlighted the need for accurate and reliable information, and it is crucial that the Ayurvedic community continues to work toward improving the standards of practice and ensuring that its treatments are based on sound scientific evidence (The Hindu, 2021; Indian Journal of Medical Ethics, 2022; Rastogi and Pandey, 2022).

Alzheimer’s disease (AD) is the leading cause of dementia globally, Rabinovici (2019) estimated to affect 44 million worldwide by 2030 (Livingston et al., 2017). At present, the mainstay treatment for AD is acetylcholinesterase inhibitors like Donepezil (Rabinovici, 2019). Though they have shown symptomatic benefits in various stages of AD, their lack of any disease-modifying effect and considerable side effects have led researchers to search for other effective and safe treatment modalities (Čolović et al., 2013; Rastogi, 2018), which led to an interest in Bacopa monnieri (Brahmi, bacopa, water hyssop) (BM). It has been used for centuries by Ayurveda (the traditional Indian system of complementary medicine), to improve memory and other cognitive domains (Singh and Dhawan, 1997).

Bacopa monnieri has shown neuroprotective effects in animals and *in vitro* studies. Human studies on patients with AD have been inconclusive. Moreover, the majority of these studies had methodologically weak designs with imprecisely used criteria for diagnosing patients, resulting in low-quality of evidence (Kongkeaw et al., 2014). We did a narrative review to elucidate the current challenges in designing clinical trials of BM in AD and identify gaps to undertake future research. We also reviewed the current issues and solutions in generating high-quality evidence regarding BM and its possible role in the treatment of dementia.

## 2. Usual challenges in conducting trials of Bacopa monnieri in Alzheimer’s disease and future directions

**FIGURE 1:**
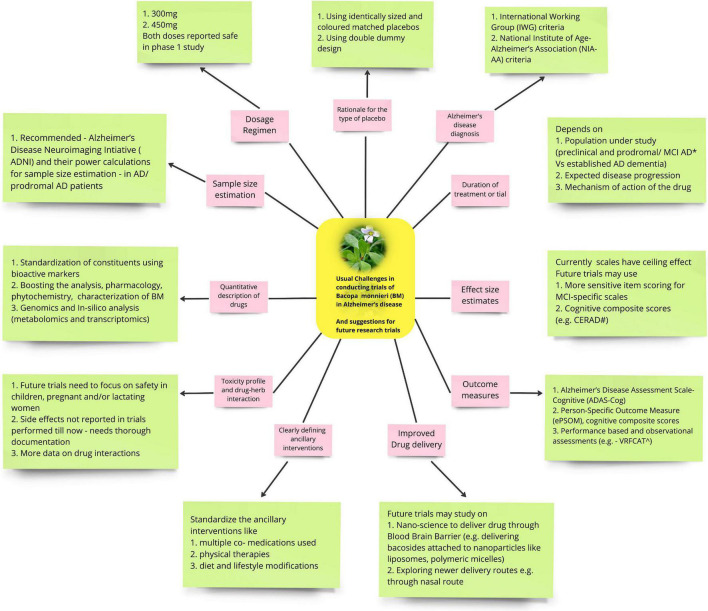
Shows the usual challenges in conducting trials of Bacopa monnieri (BM) in Alzheimer’s disease (AD) (pink boxes) and suggestions for future research trials (green boxes).

There are many factors to be considered while doing a trial of BM on AD in future (Figure 1).

### 2.1. Rationale for the type of control/placebo

Designing a placebo for herbal medications can be challenging. Herbal drugs come in various forms like powder, crystalline form, liquid or sometimes raw parts of trees, making it difficult or even impossible to have active and control drug preparations with identical color, shape, smell and taste. In the clinical trials involving BM, this concern has not yet been widely addressed in the studies conducted. BM is known to have a strong herbal taste and a bitter aftertaste. For adequate blinding in future RCTs, this issue can be circumvented by providing the active drug in capsules to be swallowed whole and using identically sized and colored matched placebo capsules (Enck et al., 2013). But this may not be practical if the drug is used in large quantities, e.g., 5–10 g or if the drug is in some other dosage form. In such situations, it would be desirable to use isolate and use the purified active component of the drug rather than its pure form.

The comparative placebo use in the evaluation of BM also raises ethical issues. It may be considered unethical to assign unaware patients a placebo instead of using an available therapy effective in treating their condition. If BM is being investigated as a disease-modifying therapy, placebo-controlled trials are required. Currently, cholinesterase inhibitors or memantine are approved symptomatic standard of care in mild to moderate AD, hence at randomization, stratification for the usage of these drugs should be done. On the other hand, If BM is being investigated as a symptomatic treatment drug, then it can either be tested against a placebo or with an acetylcholinesterase inhibitor (Gupta and Verma, 2013). Trials of BM comparing it with an existing standard of care drug can use a double dummy method to maintain the blinding. In this design, the patient has to take both the active and placebo medication (Marušić and Ferenčić, 2013; Blinding in Clinical Trials, 2023).

### 2.2. Dosage regimen

There is a wide variation in the dose of BM used for clinical trials. Kongkeaw et al. (2014) analyzed nine randomized, placebo-controlled trials and 518 patients who received >12 weeks of standardized extracts of BM. Most of the studies had used the dose of BM extract: 300 mg (about 50% bacosides) daily for 12 weeks, and therefore it became the reference point for effective dosing and treatment duration for future BM studies (Kongkeaw et al., 2014). Basheer et al. (2022) reviewed five randomized placebo-controlled trials and found considerable variation in BM dose (ranging between 125 and 500 mg twice daily). However, the effects of different doses of BM were not tested by any of those. Since both 300 mg and 450 mg of BM have been reported to be safe in a Phase 1 study, Pravina et al. (2007) future trials can evaluate both doses for efficacy and safety.

### 2.3. Quantitative description of the drugs

The use of polyherbal formulations is quite common in herbal drug trials. The CONSORT guidelines suggest that for herbal interventions, “the content (e.g., as weight, concentration; may be given as range where appropriate) of all quantified herbal product constituents, both native and added, per dosage unit form and added materials, such as binders, fillers, and other excipients” be mentioned. Special attention must be paid to the standardization of the compounds using bioactive markers. The other compounds, in addition to BM, may have a confounding effect on the outcome measures being evaluated. This was the case in the trial by Cicero et al., wherein they used a nutraceutical containing BM dry extract 320 mg, and multiple other ingredients like L-Teanina 100 mg, Crocus sativus 30 mg, some vitamins (Vitamin B6 9.5 mg, Biotine 450 mcg, Folic acid 400 mcg, Vitamin B12 33 mcg, Vitamin D 25 mcg) and Copper 2 mg, each of them having potential to influence the outcome (Cicero et al., 2017). Hence, future trials involving BM should quantify all the active/marker constituents per dosage unit form.

Another shortcoming is as per geographical locations, climatic conditions, environmental hazards, harvesting methods, and collection protocols, there may be variations in the actual dosage of the active form, making it difficult to standardize the end product for a reproducible quality (Bauer and Tittel, 1996).

Drug standardization techniques for herbal medications can also be applied to future research of BM. These include chromatographic examination, i.e., identification of the crude drug based on the use of major chemical constituents as markers; and quantitative chemical evaluation, i.e., to estimate the amount of the major classes of constituents (Garg et al., 2012; Kunle et al., 2012).

Future research should focus on boosting the analysis, pharmacology, phytochemistry, and toxicological characterization of BM. Genomics and *in silico* analysis (metabolomics and transcriptomics) should be encouraged as these can elucidate the biotechnological and therapeutic significance of the genes responsible for synthesizing active or inactive secondary metabolites of BM. Biosynthesis of bacosides also offers an opportunity to redesign its metabolic pathways either wholly or partly (Banerjee et al., 2021).

### 2.4. Clearly defining and quantifying ancillary interventions

Randomized controlled trials (RCTs) in modern medicine are largely restricted to standardized single drug/therapy interventions, while interventions using herbal drugs are often complex and personalized and usually consist of multiple medications, physical therapies, and diet and lifestyle modifications (Hankey, 2005). For instance, in addition to BM, an ayurvedic practitioner may provide relaxation techniques and other holistic approaches to patients with AD. Future trials on BM need to standardize these ancillary interventions so that the outcome measures are not biased.

### 2.5. Diagnosis of Alzheimer’s disease

Participants should be recruited into future BM trials as per internationally recognized and validated diagnostic criteria, which should be clearly defined in the study protocol. The International Working Group (IWG) criteria and the National Institute of Age-Alzheimer’s Association (NIA-AA) criteria should preferably be used as they are well-validated even for diagnosing prodromal AD (Rosenberg et al., 2021). These criteria consider three stages of Alzheimer’s disease: (1) Alzheimer’s Disease Dementia, (2) mild cognitive impairment (MCI) due to Alzheimer’s, and (3) preclinical (pre-symptomatic) Alzheimer’s (Diagnostic Criteria Guidelines).

### 2.6. Duration of treatment/trial

The trial duration depends on the studied patient population (preclinical AD and prodromal/MCI AD vs. established AD dementia). Controlled clinical trials in mild to moderate AD patients have been traditionally conducted for 6 months, but this duration may not be enough to determine clinically meaningful differences in the outcomes. It also depends on the intervention strategy planned for the study (primary vs. secondary prevention). Prevention trials require a minimum duration of 3 years.

Trial duration should also be relevant to the treatment end-points [cognition/functional/global endpoints or behavioral and psychiatric symptoms of dementia (BPSD)]. The expected disease progression rate and the assumed activity of the drug under investigation should also decide the minimum duration of confirmatory trials, e.g., in patients with mild to moderate and prodromal AD/MCI due to AD, a minimum treatment duration of 18 months has been assumed to be sufficient. In some trials, even longer studies might be necessary depending on the timing of intervention and trial design (e.g., delayed start or time-to-event approach).

The mechanism of action of the drug also affects the duration of intervention to arrive at a pre-specified outcome. For instance, if a disease-modifying drug demonstrates efficacy in MCI-AD patients, the same drug cannot be assumed to show similar efficacy when treatment is initiated at a later stage of the disease (EMA, 2018).

### 2.7. Effect size estimates and sample size and outcome measures

Currently used cognitive scales have a ceiling effect in prodromal AD/MCI as they are not sensitive enough to detect small changes in cognition. In addition, patients closer to the onset of dementia have subtle but already noticeable impairments in their daily functioning; however, the extent to which every single individual can compensate for their cognitive deficit and adjust their daily activities is very variable. The progression of the functional deficit may be difficult to gauge with currently available scales leading to difficulty in estimating sample size estimation and the power of the study.

Future studies could use more sensitive item scoring for MCI-specific scales and investigate in detail only those domains that are impaired consistently in MCI due to AD/prodromal AD so that the trial could capture the earliest clinically meaningful changes over time.

Recently cognitive composite scores [e.g., a combination of Consortium to Establish a Registry for Alzheimer’s Disease (CERAD) Word List Delayed Recall, WMS-R Logical Memory (Delayed Recall), Category Fluency, Symbol Digit Modalities Test, Ravens Progressive Matrices (9-Item), Judgment of Line Orientation (15-Item), MMSE Orientation to Time, MMSE Orientation to Place] have been developed as primary efficacy measures in AD prevention trials to detect subtle cognitive changes between treatment and placebo groups (Ritchie et al., 2017; Malek-Ahmadi et al., 2018). For AD patients, we recommend widely accepted and validated clinical outcome measures like Alzheimer’s Disease Assessment Scale-Cognitive Subscale (ADAS-Cog) for future trials.

There are several studies on power calculations of clinical trials on AD (Ard and Edland, 2011). Volumetric neuroimaging measures of the brain region such as hippocampal volume have been shown as a good surrogate marker to global cognitive scales traditionally used in clinical treatment trials. Therefore, we recommend (Alzheimer’s Disease Neuroimaging Initiative [ADNI], 2017) and their power calculations for sample size estimation for future BM trials in AD/prodromal AD patients. Effect size can be calculated as a percentage of the expected mean rate of decline under placebo/standard of care scenario. For instance, to detect a 25% decline of 2.5 points annual rate of change in ADAS-Cog score for MCI/prodromal AD (considering 80% power and two sided alpha = 0.05), the sample size needed would be 1,183 for a 1 year trial. If a more objective outcome like hippocampal atrophy on MRI or ApoE4 risk allele were used, the sample size could further be reduced. For instance, using hippocampal atrophy on MRI as inclusion criteria for 80% power with two sided alpha of 0.05 would require 25% decline of 1.5 points annual rate of change for the same effect size. Consequently the sample size would be reduced to 458 (almost half of the initial 1,183) (McEvoy et al., 2010).

Primary outcome measures in AD trials should include functional outcome measures and composite outcomes in addition to the usual cognitive measures. Introduced in 1984, Alzheimer’s Disease Assessment Scale-Cognitive (ADAS-Cog), has remained the most frequently used cognitive outcome measure in clinical drug trials for mild to moderate AD (Mohs et al., 1983). However, as discussed above, traditional scales like ADAS-Cog, Mini-Mental State Exam (MMSE) and the Clinical Dementia Rating (CDR) scale sum of boxes (CDR-SB) have a ceiling effect in detecting prodromal AD/MCI.

Prodromal AD assessment requires newer sensitive measures, rather than traditional neuropsychological tests. The electronic Person-Specific Outcome Measure (ePSOM), cognitive composite scores, prospective memory and metacognition tests are steps in this direction (Harvey et al., 2017).

Recently, performance based and observational assessments have been explored to detect functional impairment in early stages, using instruments sensitive to very subtle functional changes like tests for financial capacity, performance based skill assessments, computerized assessment procedures [e.g., Virtual Reality Functional Capacity Assessment (VRFCAT)] and direct observation measures, including video observation and ecological momentary assessment with smartphone technology (Harvey et al., 2017). These can be incorporated into future BM trials.

### 2.8. Toxicity profile and drug herb interaction

Analysis of compounds of BM, i.e., Bacoside A and B with three cytogenetic end points have been reported to have no incidence of genotoxicity (Sukumaran et al., 2019). However, toxicological studies of BM have not been adequately researched upon, especially the toxicity profile of bacosides and bacopasaponins on patient groups like children, pregnant and/or lactating women. This needs to be investigated and documented in future studies. Concomitant use of conventional anti-AD drugs and few herbs is reported to lead to clinically relevant herb-drug interactions, which needs further investigation (Gohil and Patel, 2010). Consequently, till such time, use of BM at conventional doses on patients with an underlying disease should be done with necessary precautions in order to avoid herb-medicine interaction.

Safety data is often overlooked or under-reported in trials involving *Bacopa monnieri*. In a review by Kean et al. (2017) 44% (4 out of 9 studies) of the included studies did not report any safety outcomes and tolerability data. In a recent review by Basheer et al. (2022) one of the five studies did not report the safety data. Brimson et al. (2021) similarly reported, in two of the eleven included studies, there was no information on the adverse events. A clear and unbiased description of the safety data is imperative to understand how stringently the investigators adhered to the research protocols.

### 2.9. Improved drug delivery

The blood-brain barrier (BBB) has been a great hurdle for brain drug delivery. Nanoscience has recently shown great potential in improving drug delivery to the brain across BBB. Future studies can explore delivering bacosides, the active constituent of BM, in a better concentration and in targeted areas of the brain, using nanoparticles based on liposomes, polymeric micelles, and polymersomes. Simultaneously research needs advancement to map the target areas and molecules in the brain, which can be modulated by bacoside laden nanoparticles for that specific disease. The ability of the clarifed butter to cross the blood brain barrier, could be looked into in future studies to improve delivery of BM into CNS. The combination of clarified butter and BM known as “BrahmiGhritam” is already in use in Ayurveda but it’s assumed advantage of improved CNS penetration needs further research (Chaudhari et al., 2017).

Nasya is one of the ayurvedic methods of herbal drug delivery. It involves intranasal delivery of dry herbal powders or medicated oils and more recently developed “*Geophila repens* phytosome-loaded intranasal gel” (MEGR-PG) (Rajamma et al., 2022). Investigators may evaluate the safety followed by efficacy of this route, if it can actually achieve any effective therapeutic concentration in the CNS. If this route is found to be safe and efficacious, further clinical trials may be performed and extended to trials of BM in AD.

## 3. Generating high quality evidence regarding any possible role for BM in treatment of dementia

Although neurodegenerative dementia is quite common, there is still no cure yet and conventional treatments are often limited. Many patients therefore turn to complementary and alternative medicine (CAM). Due to increased popularity, research on the benefit of BM are growing, especially for treating “memory loss” (Wells et al., 2010). However, the ethical aspects of trials involving alternative and complementary medications have not been rigorously conducted, but they are popular among the users as they are supposed to have historical evidence supporting their use and that these therapies are “natural” and hence “safe” (Miller et al., 2004).

**FIGURE 2:**
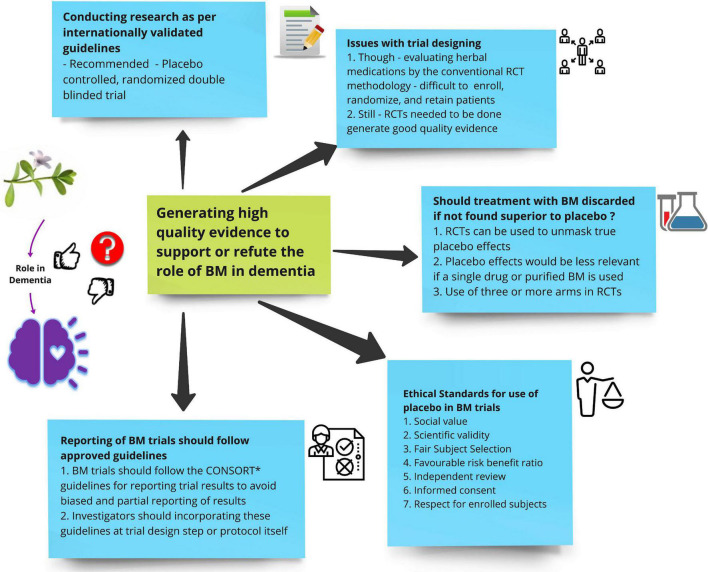
Shows suggestions for future investigators on how to generate high-quality evidence to support or refute the role of Bacopa monnieri (BM) in dementia.

Herbal medications may produce adverse effects and may also modify the effect of conventional medications. For instance gingko biloba and cranberry have been known to increase the risk of bleeding in patients already on anti-platelets and anti-coagulants (Sagers, 2010). Additionally patients with cognitive impairment may be susceptible to poor judgment given their condition, so appropriate counseling about treatment and recommendations becomes critically important. It’s high time rigorous and qualitative research are conducted to establish level I evidence for the use of BM in dementia (Figure 2).

### 3.1. Importance of conducting research as per internationally validated guidelines

A noticeable increase in the use of CAM has led physicians to seek reliable, evidence—based information for both personal and professional use. The same holds true for BM in the treatment of dementia. The widespread usage of BM underpins the need to conduct large scale, preferably RCTs to irrevocably establish the assumed safety and efficacy of BM in treating dementia.

Data obtained from adequately powered research methodology is vital to the knowledge about the risk-benefit ratio of BM in dementia patients, which in turn will contribute to evidence-based medicine and help physicians in patient management decisions.

Bacopa monnieri has many active ingredients and only through research can the mechanisms of action of pertinent ingredient can be elucidated. Moreover, BM is readily available in pharmaceutical outlets and can be procured as over the counter drug also. Hence the availability of concrete evidence becomes more important for consumers to make informed personal healthcare choices and limit the indiscriminate use of unsafe or expensive or ineffective therapy.

Performing research as per validated guidelines will lead to selection of subjects in accordance with primary ethics cleared objectives of the study and prevent unwanted exposure to vulnerable patient groups. Quality of RCTs conducted on CAM has long been debated. In a systematic review of more than 200 RCTs on CAM showed that most trials did not mention how the random sequence was generated, inadequately concealed the allocation sequence, and did not use intention -to- treat analysis. These shortcomings often lead to overestimation of positive treatment effects (Egger et al., 2003).

Similarly in a systematic review involving five trials on the effects of BM on AD, Basheer et al. (2022) showed that there was a high risk of bias in most of the studies with regards to assessing the primary outcome measures and missing data and assessment of lost to follow-up data. Additionally, there was considerable heterogeneity in the outcome assessment in the trials included (Basheer et al., 2022). This hampers the generalizability of the results of the trials and the level of evidence obtained also gets downgraded.

The placebo -controlled, randomized double blinded trial is internationally accepted as the most rigorous clinical design to adjudicate the correct risk-benefit ratios of therapies. These standards have recently been extended to RCTs of CAMs (Gagnier et al., 2006). Some commentators have presented a different argument that CAM rely heavily on a “holistic personalized” approach to treatment which would be difficult to replicate in the rigors of randomized placebo controlled trials (Gautama, 2021).

Some have advocated ayurveda-appropriate trial models are to overcome these shortcomings like the N-of-1 trial, stepped wedge design and “black box” model (Singh and Dhawan, 1997). Though these arguments may hold true in the context for CAM like acupuncture or healing ritual and mind-body therapy, herbal interventions like BM, wherein the active ingredient is a biochemical constituent, can undergo the rigors of placebo—controlled RCTs.

Bacopa monnieri constituents a major component of polyherbal formulations that are procured by users from Ayurvedic or general medicine stores, mostly without a valid prescription. This precludes the concern that RCTs of BM would alter its therapeutic milieu on dementia treatment. Therefore, there is no reason why BM or any other herbal remedy should not be evaluated in accordance with the same rigorous standards as conventional drugs.

### 3.2. Issues with trial designing

Further concern in evaluating herbal medications by the conventional RCT methodology is the difficulty in enrolling, randomizing, and retaining patients. Patients who prefer herbal medications may decline to participate in blinded RCTs as they may not prefer to be assigned to a comparator group receiving conventional medications or a placebo. Likewise, patients who have a strong preference for conventional medications could be reluctant to try herbal medications like BM. This may potentially impede generalizability of the trial results and restrict patient participation. Furthermore, considering the easy availability herbal medications sometimes even as OTC drug in the open market, the control group can easily cheat and subsequently confound the trial outcomes.

Though retrospective or prospective cohort studies can provide valuable data with regards to outcomes measures, they cannot measure treatment efficacy. Additionally, in trials involving dementia, a high rate of placebo response is known to happen (Jeste and Lee, 2017). Thus clinical trials evaluating BM for dementia, would lack scientific validity, without the use of placebo controls, making interpretation of results challenging or sometimes impossible. The evidence based information acquired through rigorous research comparing BM to placebo or other conventional medications would not only help CAM practitioners make informed decisions for their patients but also the patients themselves can make an informed choice for themselves, as most insurance companies do not pay for these CAM therapies and they have to pay the entire amount out-of-pocket (NCCIH, 2016).

### 3.3. Ethical standards for use of placebo in BM trials

Enrolling patients to placebo groups in RCTs for diseases where a proven efficacious or symptomatic treatment is available, raises ethical issues. Emanuel et al. (2000) had suggested seven fundamental principles to help guide trial investigators design placebo-controlled trials in an ethical manner. In this section, we try to explore how these principles can be applied to future dementia trials using BM.

#### 3.3.1. Principles

a. Social value–trials of BM in dementia must be tailored to cater to a broader population group and research should be able to generate information that will have the potential to improve human health.
b. Scientific validity—Placebo controlled double blinded randomized trials are considered as best clinical design to demonstrate valid treatment efficacy. As discussed above, in trials involving BM, the difficulty with use of placebo has not yet been specifically mentioned in trials already conducted, still in future, trials can circumvent the issue by using opaque bland capsules to mask the bitter taste of BM. Additionally, for treatment of neuro-degenerative dementia, there is no cure as yet developed, however, acetylcholinesterase group of drugs like donepezil, are approved drugs for symptomatic treatment. Therefore, using BM active arm and a placebo or the current symptomatic treatments as control group should not possess a significant ethical issue.
c. Fair Subject selection—The sample population should be relevant to the population who will receive the intervention in a “real world” scenario. Investigators should avoid preferential selection of patients in BM trials based on expected trial outcomes. Literature till now is inconclusive on the definite benefit of BM in improving cognition in test subjects especially in the domain of memory. Hence, investigators should not be biased into preferentially enrolling vulnerable patients to BM or privileged patients into other conventional trials of BM or vice versa. Priority should be given to achieving the research’s scientific goals, with risk mitigation, value enhancement, and efficacy measurement.
d. Favorable risk benefit ratio–BM in the previous trials have not been reported to have significant side-effects. However, as discussed above the toxicological studies of BM have not been adequately researched upon, especially the toxicity profile of bacosides and bacopasaponins on patient groups like children, pregnant and/or lactating women. Moreover, the risks of placebo treatment should be minimized by a careful adjudication of the duration of placebo treatment so that prolonged duration of placebo use does not pose undue risks of serious harm or discomfort to the patients.
e. Independent review—This principle applies to all clinical trials and not only to BM. Independent review safeguards the participants from unethical, unwarranted deviations from the pre-approved protocols and also maintains vigilance over investigators claims about the scientific need of the placebo and has no unwarranted risks.
f. Informed consent—Patient and their caretakers should be explained in details about the active drug and also the use of placebo and the risks of being enrolled in either arm. Especially in case of trials involving dementia patients where the patient may not be cognitively coherent to make an informed choice about study participation, hence the importance of acquiring a valid informed consent from the next of kin before enrolling the patient becomes all the more important.
g. Respect for enrolled subjects—Investigators should ensure patient data are kept confidential, any unexpected or expected adverse events be attended to and/or rightfully compensated, informing participants of any newly detected risks or benefits of the therapy under investigation, flexibility in permitting patient withdrawal as and when necessary and keeping a third-party surveillance. If patient with dementia, enrolled in trial is later found to have a reversible cause of dementia, for instance immunologically mediated dementias, Rosenbloom et al. (2009) the subject should be promptly withdrawn from trial and appropriate treatment instituted. The trials participants also have the right to be informed about the results of the trial.

### 3.4. Should treatment with BM discarded if not found superior to placebo?

Complementary and alternative medicine therapies have primary been used to treat chronic conditions in addition to conventional therapies. The debate continues whether patients should be denied these treatment which are of partial effectiveness or of low risk, because the treatment could not show itself as having superior therapeutic efficacy as compared to placebo. When patients do not have any better alternative to their ailment, or the available conventional therapies have significant or distressing side-effects, should they also be denied the consolation of “enhanced placebo effect”? (Kaptchuk, 2002). With regard to this, some commentators have advocated that the usual holistic approach used in CAM and the comprehensive interaction between the patients and practitioners may lead to increased possibility of placebo effect. True placebo effects can be distinguished using RCTs that compare a treatment with a placebo control and a no-treatment group. The number of study arms in a clinical trial can affect the ability of researchers to determine the effect size of the placebo and drug response. In a clinical trial with a larger number of study arms, it is possible to compare the response to the drug and the response to the placebo more accurately. This is because a larger number of study arms allows for better control of confounding variables that may influence the results of the trial (Enck et al., 2011).

The placebo effects in CAM treatment have been observed more with procedures like acupuncture and ritualistic healings (Smith et al., 2002). However, when a single drug like BM or its active ingredient is under evaluation, the placebo effect is less relevant. RCTs employing three or more arms showing patients randomized to BM treatment having better outcomes compared to those randomized to no treatment, even though the treatment is no better than placebo, would generate the most compelling evidence for the treatment efficacy of BM in dementia. Additionally, if trials are using a holistic approach, for instance combining ritualistic healing in addition to BM, the importance of demonstrating the superior treatment efficacy of BM compared to placebo should not be undermined by efforts to prove the placebo response of ritualistic healing.

### 3.5. Reporting of BM trials should follow approved guidelines

As discussed above also, reporting in clinical trials of BM lacked completeness regarding information on all the necessary outcome measures and missing data handling (Kongkeaw et al., 2014; Basheer et al., 2022). Partial and biased reporting of trial results after completion leads to difficulty in evaluating the quality of evidence and also retrieving pertinent outcome data for a meta-analysis becomes challenging. A number of initiatives have been made to standardize trial reports in order to improve reporting. The CONSORT tool is the internationally accepted tool of choice for reporting results of RCTs. Recently these have been extended to CAM, which have been briefly discussed in the section below. Apart for trial sponsors and investigators, journal editors also have a moral obligation to not consider manuscripts which do not follow these guidelines in reporting results.

Additionally, this will motivate the investigators to consider these guidelines at the time of planning the trial design itself, incorporate them into the research protocol, and conduct the trial accordingly, so that the overall quality of investigation and the evidence obtained from it gets upgraded.

## 4. Conclusion

Future studies on BM should implement more RCTs with appropriate sample size of accurately diagnosed AD/prodromal AD patients, administering recommended dosage of BM and for a pre-specified time period calculated to achieve adequate power for the study. Researchers should also try to develop and validate more sensitive cognitive scales, especially for prodromal AD. BM should be evaluated in accordance with the same rigorous standards as conventional drugs to generate best quality evidence.

## Author contributions

BM and VV: conceptualization. BM, AA, and VV: methodology. AA and BM: writing—original draft preparation. BM, AA, AG, MS, AB, JS, and VV: writing—review and editing. AG and MS: supervision and writing—review and editing. All authors have read and agreed to the published version of the manuscript.
